# Composting-Like Conditions Are More Efficient for Enrichment and Diversity of Organisms Containing Cellulase-Encoding Genes than Submerged Cultures

**DOI:** 10.1371/journal.pone.0167216

**Published:** 2016-12-09

**Authors:** Senta Heiss-Blanquet, Françoise Fayolle-Guichard, Vincent Lombard, Agnès Hébert, Pedro M. Coutinho, Alexis Groppi, Aurélien Barre, Bernard Henrissat

**Affiliations:** 1 Department of Biotechnology, IFP Energies nouvelles, Rueil-Malmaison, France; 2 Architecture et Fonction des Macromolécules Biologiques, CNRS, Aix-Marseille Université, Marseille, France; 3 Centre de Génomique Fonctionnelle de Bordeaux, Université de Bordeaux, Bordeaux, France; 4 INRA, AFMB, Marseille, France; 5 Department of Biological Sciences, King Abdulaziz University, Jeddah, Saudi Arabia; Austrian Institute of Technology, AUSTRIA

## Abstract

Cost-effective biofuel production from lignocellulosic biomass depends on efficient degradation of the plant cell wall. One of the major obstacles for the development of a cost-efficient process is the lack of resistance of currently used fungal enzymes to harsh conditions such as high temperature. Adapted, thermophilic microbial communities provide a huge reservoir of potentially interesting lignocellulose-degrading enzymes for improvement of the cellulose hydrolysis step. In order to identify such enzymes, a leaf and wood chip compost was enriched on a mixture of thermo-chemically pretreated wheat straw, poplar and *Miscanthus* under thermophile conditions, but in two different set-ups. Unexpectedly, metagenome sequencing revealed that incubation of the lignocellulosic substrate with compost as inoculum in a suspension culture resulted in an impoverishment of putative cellulase- and hemicellulase-encoding genes. However, mimicking composting conditions without liquid phase yielded a high number and diversity of glycoside hydrolase genes and an enrichment of genes encoding cellulose binding domains. These identified genes were most closely related to species from *Actinobacteria*, which seem to constitute important players of lignocellulose degradation under the applied conditions. The study highlights that subtle changes in an enrichment set-up can have an important impact on composition and functions of the microcosm. Composting-like conditions were found to be the most successful method for enrichment in species with high biomass degrading capacity.

## Introduction

Lignocellulosic biomass is a worldwide abundant raw natural material which has gained enormous interest as substrate for environmentally friendly and sustainable biofuel production. Composed of cellulose, hemicelluloses, pectins and lignin, it is nevertheless a very complex and heterogeneous substrate, and thus very resistant to natural degradation. The carbohydrate constituents (cellulose, pectins and hemicelluloses) are embedded in the hydrophobic lignin matrix that severely limits access to degradation enzymes. Consequently, the enzymatic decomposition step is slow and incomplete, hampering the development of cost efficient production processes for biobased products including biopolymers, chemicals and biofuels [1,2]. To overcome these difficulties, intense research is presently addressing the identification of ever more efficient and robust enzymes for saccharification of cellulose to fermentable sugars.

In nature, lignocellulosic biomass can be degraded by archaea, bacteria and fungi. Due to the complex composition of lignocellulosic biomass, a large spectrum of enzymatic activities is required for its complete degradation. Microorganisms produce a mixture of cellulases, hemicellulases, pectinases and lignolytic enzymes that act in concert to extract the necessary nutrients from the plant biomass. These enzymes, generally termed as “Carbohydrate Active enzymes” (CAZymes) and classified in sequence-based families in the CAZy database [3], are expected to cover the whole scope of complementary activities needed for this degradation. Furthermore, the enzymatic cocktail produced by any given organism is adapted to its ecological niche and to the substrates present in its habitat. Biomass-degrading microorganisms also often act in synergy in order to optimize their growth efforts by minimizing the energetic expense for production of the required degradation enzymes and by maximizing benefits of their common action. For these reasons, metagenomic studies focusing on whole microbial communities rather than isolated organisms, are more likely to identify interesting enzymes for lignocellulose degradation, especially when these communities are derived from specialized communities from lignocellulose-rich ecological niches, such as those from soil, or from specific natural or artificial environments such as compost, or the digestive tract of large herbivores or termites [4–7].

To obtain still more efficient and better-suited enzymes, very recent studies have attempted to specifically adapt natural communities to industrially relevant substrates by hypothesizing that species encoding enzymatic activities most relevant for industrial biomass degradation would be enriched [8–11]. In this case, the catabolic potential can be specifically adapted to industrially prepared substrates, which may be thermochemically pretreated and require different enzymatic mixtures for its efficient degradation. Another advantage of such enrichments is the reduction of the microbial diversity when compared to the original natural habitat. This greatly facilitates the analysis and increases the probability of detecting relevant genes [12]. Recent studies have shown a high content in glycoside hydrolases [13,14] or an increase in endoglucanase or endoxylanase activities after incubation of compost samples on lignocellulosic substrates, such as switchgrass and rice straw [10]. Concerning dominating taxa after enrichments, different origins of microbiota and different composting conditions often make comparisons and common conclusions difficult. Thus, *Proteobacteria* were found to accumulate in some cases [15,16], whereas *Actinobacteria* or *Firmicutes* were found to dominate in other [10,14]. These results, although giving insight into the complex taxonomic composition and functional potential of such enrichments, demonstrate the difficulty to pinpoint the importance of special taxonomic units for degradation of a given lignocellulosic substrate, especially as the genetic potential related to biomass degradation of the compost community before the enrichment was not analyzed. In consequence, functions that specifically appear upon exposure to the new substrates are more difficult to identify. In addition, the influence of enrichment conditions other than temperature have rarely been investigated.

In the present study, we performed enrichment cultures of a compost sample on a mixture of industrially relevant lignocellulosic substrates under thermophilic conditions. Thermophilic microorganisms possess highly efficient enzymes for biomass degradation which often also display improved resistance to harsh process conditions including the presence of solvents and of inhibiting compounds. This feature is interesting for industrial applications which generally make use of enzymes from mesophilic fungi such as *Trichoderma reesei* for biomass degradation. Although being secreted at high levels, they are rather sensitive to high temperatures or the presence of inhibitors such as phenol derivates [17], whereas bacterial enzymes tolerate a higher range of process conditions.

In order to identify relevant players for lignocellulose decomposition among the bacterial thermophiles and investigate the impact of the substrate on the perturbation of the original compost microflora, the metagenomic sequence of both the initial compost and enrichments were determined. Furthermore, different enrichment set-ups were conducted, which showed that culture conditions shaped community structure and function. Data collected by 454 pyrosequencing allowed detailed functional and taxonomic analyses of all microcosms. The analyzed consortia differed in the total number and diversity of glycoside hydrolases and of other CAZymes, which were found to originate from different bacterial orders in each case. The impact of each condition on the observed changes in the microbiome composition and the lignocellulose degradation potential is discussed.

## Materials and Methods

### Enrichment cultures

Compost samples were collected at a municipal compost platform from a thermophilic pile composed of leaves and chopped wood residues. The permission for sampling was issued by the company Bio Yvelines Service. Samples were taken at a depth of about 20 cm where the temperature was about 50°C. The samples were incubated with a lignocellulosic substrate (LCB) consisting of equal parts of three different pretreated substrates: dilute acid pretreated *Miscanthus giganteus*, wheat straw and poplar which were both steam-exploded without addition of acid. All substrates were washed three times to remove soluble compounds and dried. The composition of lyophilized substrates (Table 1) was determined using the Van Soest procedure ([18], Invivo Labs, Château-Thierry, France). Compost and substrates were mixed at a 1:4 ratio (dry weight basis) and incubated in glass flasks of 5.7 l. In the liquid culture, the dry matter was suspended at 5% (w/v) in a total volume of 300 ml of a mineral medium (12.8 gl^-1^ Na_2_HPO_4_.7H_2_O, 3 gl^-1^ KH_2_PO_4_, 0.5 gl^-1^ NaCl, 1 gl^-1^ NH_4_NO_3_, 2 mM MgCl_2_, 2 mM CaCl_2_, pH 6.5). Solid cultures were adjusted to 60% humidity and a C:N ratio of 20:1 with NH_4_NO_3_. All enrichment cultures were incubated at 55°C for seven months. The liquid cultures were moderately agitated (100 rpm) whereas solid cultures were static, and the flasks shaken manually every three days. As a control, the compost sample alone was also incubated, both in suspension and as a solid culture. [19]. To avoid oxygen limitation, flasks were aerated for one hour under sterile conditions when CO_2_ concentration reached 10% (v/v).

**Table 1:** Composition of pretreated lignocelluloses substrates, expressed as percentages of dry weight.

	lignin	cellulose	hemicellulose
Miscanthus	19.0	53.1	1.5
wheat straw	7.9	52.7	12.9
poplar	16.2	57.4	10.9

### DNA extraction and sequencing

DNA was extracted using an indirect extraction procedure. In order to obtain high quality prokaryotic DNA, microorganisms were first separated from the lignocellulosic biomass by an overnight incubation of 1 g dry weight equivalent in 10 ml of a biofilm disruption buffer (saline solution containing 1% methanol, 1% *ter*-butanol and 0.1% Tween 80 at 4°C [20]). The sample was vortexed for 1 min, decanted and the solid residue re-extracted twice with 5 ml of the same buffer and once with 10 ml 0.9% NaCl. Cells from all supernatants were collected by centrifugation, washed once with saline solution, and finally resuspended in 20 ml 0.9% NaCl. Bacteria were further purified by a Nicodenz density gradient [21]. 7 ml of Nicodenz solution at 1.3 g ml^-1^ were delicately pipetted below the cell suspension and centrifuged at 10,000g for 40 minutes. Bacterial cells that had accumulated on top of the density gradient were carefully harvested, centrifuged and washed with 0.9% NaCl. The bacterial cell pellet was transferred to a microcentrifuge tube for cell lysis and extraction by a protocol adapted from Aljanabi and Martinez [22]. Briefly, 1 ml of extraction buffer (0.4 M NaCl, 10 mM Tris-HCl pH 8, 2 mM EDTA) was added to the cells. The suspension was incubated with 5 mg ml^-1^ lysozyme and 1 μl DNAse free RNAse for one hour at 37°C. 100 μl of 20% SDS and 80 μl proteinase K (25 mg ml^-1^) were added and the mixture incubated one hour at 55°C. 750 μl of 6 M NaCl was added and mixed well by inverting the tubes for 30 seconds. Cell debris and impurities were eliminated by a two-step centrifugation at 20,000g for 30 min and another 10 min. After adding 1 volume of isopropanol, DNA was precipitated by incubation at -20°C for 1 hour, centrifuged at 20,000g for 30 min, washed and resuspended in 100 μl of TE. Finally, DNA was purified by the Agencourt AMpure XP kit (Beckman Coulter, Indianapolis, USA).

Preparation of shotgun libraries, amplification by emulsion PCR and sequencing was performed by Eurofins (Ebersberg, Germany) using the Roche 454 GS FLX++ technology.

### Enzymatic activity measurements

400 mg of the solid residues from enrichment cultures or initial compost were incubated in 20 ml of mineral medium containing 50 mg of LCB substrate at 55°C. After 11 days, when CO_2_ development monitoring confirmed active degradation, 800 μl of each supernatant or whole slurry was taken for the enzymatic assays. Activities were measured in a 1:1 mixture containing 2% substrate (carboxymethylcellulose or birch wood xylan, respectively) and 0.05% sodium azide in a 100 mM sodium acetate buffer (pH 5) at 55°C. The reaction was stopped at different time points between 15 min and 6 hours by boiling the samples for 5 minutes. Soluble sugars were determined by the DNS method [23]. Time points during the linear phase were used for activity calculation. At least three independent measurements were conducted with each sample.

### Metagenome sequencing, assembly and annotation

Sequence data obtained by 454 metagenome pyrosequencing were submitted to the MG-RAST server ([24], metagenome names: CI_AllReads_clip, LCBS_1_AllReads_clip, LCBS_2_AllReads_clip, LCBL_1_AllReads_clip, LCBL_2_AllReads_clip, LCBLbis_AllReads_clip, IDs mgm4544115.3, mgm4544116.3, mgm4522117.3, mgm4544118.3, mgm4559243.3, mgm4559244.3). NGS reads were quality-checked using the NGS QC Toolkit (v. 2.3.2). The threshold for retaining a read was fixed at an average quality beyond 20 for 70% of the sequence. Considering the sequencing technology and its bias, sequences were trimmed on the right end if homopolymers of at least 6 bases were present. Highly redundant sequences were removed in order to decrease the sample size, eliminate errors and facilitate assembly. This technique, known as « digital normalization », was implemented using the « normalize-by-median » script provided by the khmer software (v1.0) [25], with an abundance threshold of 5 (meaning that sequences displaying a median k-mer abundance below the specified cutoff (here cutoff = 5) are discarded). The resulting normalized high quality reads were finally assembled to contigs using CLC, Metavelvet and Abyss assemblers (v 1.5.1) with a K-mer size of 51. To circumvent typical assembly issues, such as chimerical contigs that impact gene prediction, genes were determined directly from reads using FragGeneScan. This approach, based on the EBI metagenomic pipeline (https://www.ebi.ac.uk/metagenomics/pipelines/2.0), allows to predict gene fragments which are then annotated by comparing them to the Uniprot (using BLAST) and TIGRFAM (using HMM_SCAN) databases. Functional annotation of genes was performed by considering all pertinent homologs (BLAST) and protein family assignments (HMM_SCAN) in order to provide an annotation as precise as possible while avoiding classical over-interpretation. Standardized annotation terms (GO terms and functional categories), useful for comparison, were extracted from these annotations. The number of genes in each functional category was normalized to the total number of annotated genes for each metagenome. Taxonomic analysis was conducted with high quality reads using phylosift [26]. Principal component analysis and rarefaction curves were created using the MG-RAST server.

### Analysis of carbohydrate-active enzymes encoding genes

High quality reads and assembled contigs were submitted to CAZy annotation. A sequence library was generated from the entries listed in CAZy database (www.cazy.org [3]) corresponding to glycoside hydrolases, glycosyltransferases, polysaccharide lyases, carbohydrate esterases, auxiliary activities and carbohydrate-binding modules. This library was supplemented by sequences of cellulosomal cohesins and dockerins extracted from the literature. Reads and assembled contigs were analyzed using FASTY(V35) [27]. Hits were retained when they had an e-value better than 10^−6^ with at least 30% sequence identity to at least two sequences in the library.

### Statistics analysis

Metagenome sequencing generated a first set of about 500.000 454 reads (40 Mb) for the three samples CI (initial compost), LCB_s (solid-state culture) and LCB_l (submerged culture), leading to data sets CI, LCB_S1 and LCB_L1. After rarefaction curves confirmed good diversity coverage, the impact of enrichments was then confirmed by a second sequence data set of equal size for LCB_s and LCB_l samples (data sets LCB_S2 and LCB_L2). In order to evaluate the reproducibility of the impact of enrichment conditions, a sample from a replicate suspension culture (LCB_lr) was also sequenced (data set LCB_Lr). A second sequencing run was not done for this enrichment, as the technical replicates for LCB_s and LCB_l cultures showed nearly identical results. For comparing hit numbers of individual CAZyme families and functional classes, the contribution cnt (Ф^2^) of each selected enzyme family to the total number of important CAZymes involved in lignocellulose degradation was determined. Ф^2^ is calculated from the contingency table where n_ij_ equals the number of representatives of a given family (i) present in a given sample (j) ([28]). $n_{i.}=\sum_{j=1}^{j=J}n_{ij}$ represents the sum of enzymes of one family in j samples and $n_{.j}=\sum_{i=1}^{i=I}n_{ij}$ the sum of occurrences of all enzymes present in a sample j. Finally, n_.._ = ∑_i_∑_j_ n_ij_ is the total number of enzymes in all samples. Ф^2^ is calculated as:

$$\phi^{2}=\sum_{i,j}\frac{{\left(f_{ij}-f_{i.}f_{.j}\right)}^{2}}{f_{i.}f_{.j}}$$ (1)

with $f_{ij}=\frac{n_{ij}}{n_{..}}$ being defined as the probability that an enzyme representative i is present in j and $f_{i.}=\frac{n_{i.}}{n_{..}}$ and $f_{.j}=\frac{n_{.j}}{n_{..}}$ the marginal probabilities with respect to enzyme classes i and samples j.

From (1) we can define the contribution of each enzyme class i and sample j to Ф^2^ as:

$$cnt{\left(\phi^{2}\right)}_{ij}=100.signe(f_{ij}-f_{i.}f_{.j})\frac{\phi_{ij}^{2}}{\phi^{2}}$$ (2)

This value is high if a strong dependency exists between i and j. A positive value means a high proportion of the enzyme class i in the sample j and, inversely, a negative one means a low proportion of i.

The significance of contribution of each class was checked by bootstrap analysis of the Ф^2^ data matrix by randomly selecting enzyme classes ([29]). Results were displayed as boxplots to visualize differences in enzyme counts between replicate sequence sets and samples (see supporting information). Differences were considered as significant if the boxes corresponding to 75% of the variability did not overlap and if the variation was not higher between the biological LCB_l and LCB_lr replicates than between the two LCB_s sequencing runs.

## Results

### Set-up of enrichments on lignocellulose

A mix of lignocellulosic material was inoculated with the compost microflora: the mix contained equal parts of pretreated wheat straw, *Miscanthus* and poplar (termed “LCB” substrate). These three substrates were chosen, as they represent the most relevant lignocellulosic resources currently considered for second generation biofuel production in France. The composition of the substrates is indicated in Table 1. *Miscanthus* had a low percentage of hemicellulose, as most of it was removed by the dilute acid pretreatment. In contrast, steam-explosion of wheat straw and poplar was conducted without acids, thus preserving most of the hemicellulose fractions.

**Fig 1:**
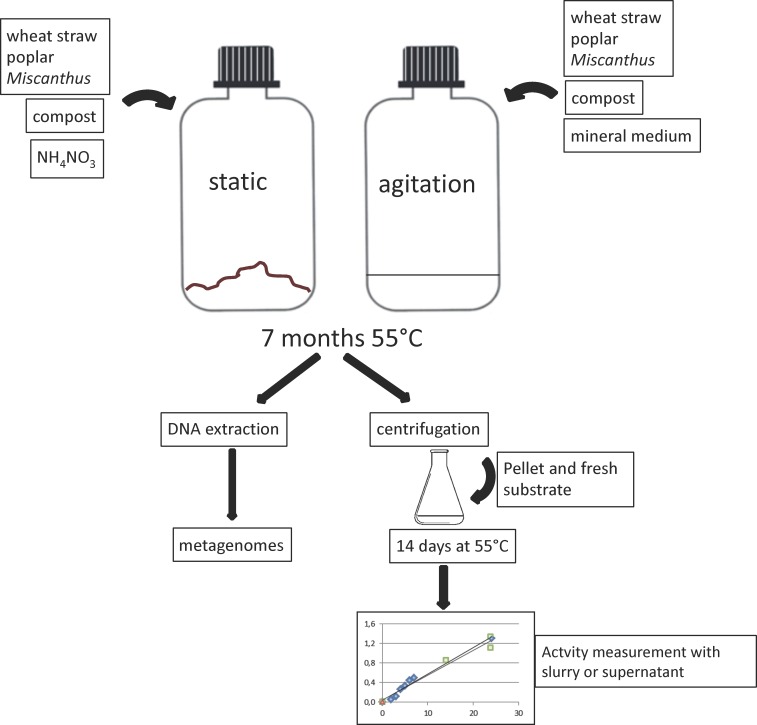
Schematic representation of experiment set-up and workflow of sampling for DNA extraction and activity measurements

Two different set-ups were devised (Fig 1): The substrate was incubated with the compost sample either in a submerged culture under agitation, thus facilitating the access of microorganisms to their substrate and assuring a good repartition of the nutrients that are liberated from the insoluble substrate. In the second case, the solid substrate was mixed with the compost and humidified, in order to mimic composting conditions. These latter cultures were static and only mixed manually every three days. Metabolic activity was evidenced by regular CO_2_ measurements in all test flasks and the control flasks. No significant CO_2_ production could be measured in the latter, suggesting that the CO_2_ generated in the enrichment cultures was due to degradation of the lignocellulosic substrate.

CO_2_ development only slightly declined after seven months, suggesting that substrate degradation was still ongoing. At that time, significant amounts of biomass was still present in all cases after that time, but substrate analysis indicated that both cellulose and hemicelluloses had been degraded. A slightly higher degradation of polysaccharides was observed for the solid state culture, but was far from complete in both enrichments (not shown). In order to assess the metabolic activities present in the enrichments, endoglucanase and xylanase activities were determined for the two cultures and the initial compost sample (Table 2). To this end, solids were incubated in mineral medium with LCB substrate, and activities determined with either the supernatant or the whole slurry, thereby distinguishing between free and substrate bound enzymes. For all samples, CMCase activity which is essentially due to endoglucanases was higher when insoluble fractions were present, indicating that a substantial part of cellulases are attached to either the substrate or the cell wall. In addition, CMCase activity was significantly increased in the solid enrichment LCB_s compared to the initial compost, but not in the suspension culture LCB_l. However, xylanase activities were very high in the initial compost sample, but were much lower in both enrichments. Interestingly, in the two enrichment cultures these activities seemed to be associated to the insoluble substrate (similarly to cellulase activities), but this was not the case for the initial compost sample where xylanase activities were not significantly different between the supernatant and the whole slurry.

**Table 2:** Endoglucanase and xylanase activities (nmol min^-1^ ml^-1^) of initial compost sample and enrichments.

	endoglucanase		xylanase	
	SN	Whole slurry	SN	Whole slurry
LCB_s	7.7 ± 2.1	14.0 ± 0.7	4.5 ± 0.4	11.8 ± 2.5
LCB_l	1.8 ± 0.6	5.9 ± 1.5	4.4 ± 0.7	12.0 ± 3.3
CI	2.2 ± 0.8	6.9 ± 1.7	46 ± 8	56 ± 13

Activities of either the supernatant (SN) or the whole slurry were determined after incubation of the solids in liquid medium containing 0.25% LCB substrate during two weeks. Values are means of at least three independent measurements.

### Taxonomic analysis

**Fig 2:**
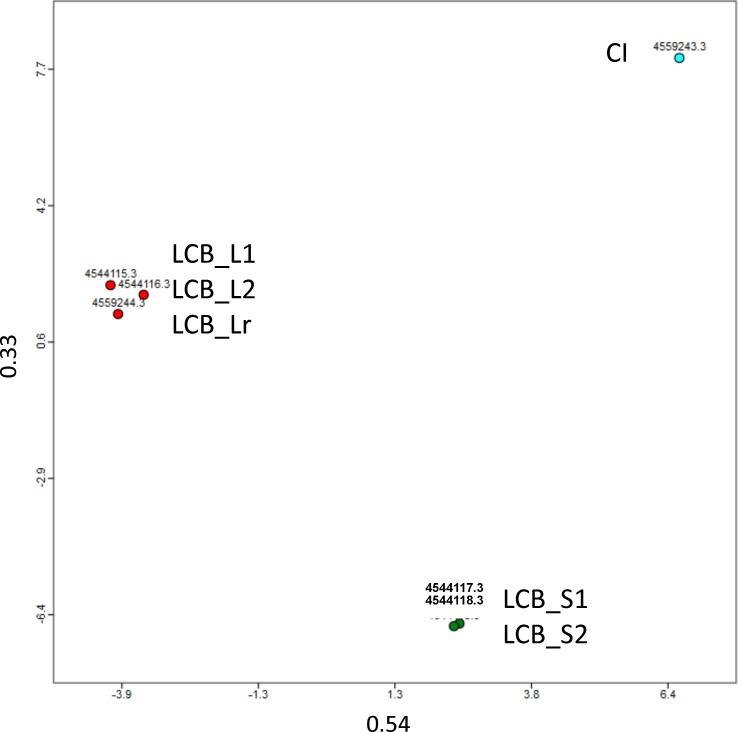
Principal component analysis of original compost metagenome CI (blue), LCB_S1, LCB_S2 (green), LCB_L1, LCB_L2 and LCB_Lr metagenomes (red), realized with MG-RAST. The two components represented explain about 90% of the difference between the samples.

Metagenomic DNA was extracted and sequenced by 454 pyrosequencing. The rarefaction curve (S1 Fig) showed that with about 500,000 reads and a total sequencing length of about 370 to 480 Mb (Table 3), most of the diversity could be covered for all samples. Not surprisingly, the initial compost sample CI displayed a higher diversity than the enrichments. Principal component analysis of all four data sets confirmed that initial compost, solid and liquid enrichments were significantly different, but that the results obtained with replicate sequencing runs and/or replicate cultures LCB_S1 and LCB_S2, as well as LCB_L1, LCB_L2 and LCB_Lr were reproducible (Fig 2).

**Table 3:** Summary of raw and assembled data obtained in the six 454 pyrosequencing runs.

	Number of reads		Mean length	Total length (Mb)	Total length assembled (Mb)	Total length in contigs	N° singletons	bp singletons	GC content (%)	Total contigs	Contigs >500 bp	N50	Largest contig
	total	retained											
CI	645,975	562,927	740	480	82.0	15.6	11047	6839737	55.9	40,492	12,159	627	5,123
LCB_S1	639,740	540,312	552	446	93.9	18.8	9571	4706385	54.7	44,255	12,916	707	8,937
LCB_S2	643,843	539,537	562	465	89.3	17.9	7902	4151628	54.0	43,313	12,114	693	13,508
LCB_L1	592,278	537,124	610	370	123.0	23.1	5102	2584593	65.0	34,004	12,978	1433	18,184
LCB_L2	561,206	504,096	686	386	122.0	23.0	5332	2952380	65.2	31,552	12,209	1588	20,684
LCB_Lr	567,104	516,243	632	367	97.3	18.6	3219	1680862	66.4	28,828	11,072	1296	12,682

Reads were assembled using the ABySS algorithm. CI (initial compost sample), LCB_S1, LCB_S2 (solid culture) and LCB_L1, LCB_L2, LCB_Lr (suspension cultures).

Assembly was performed using three different algorithms (ABySS, CLC and Metavelvet). Results obtained with ABySS showed the best trade-off between contig length and ratio of over-assembly and thus this procedure was applied for all assemblies (S1 Table).

**Fig 3:**
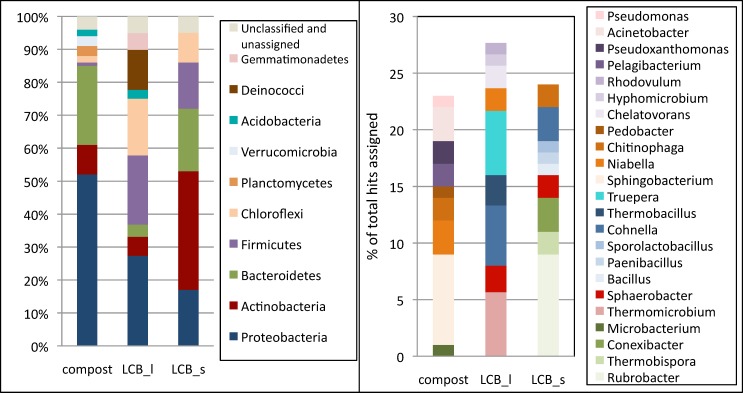
Taxonomic analysis of compost, LCB_l, LCB_lr and LCB_s cultures on a phylum level (left panel) and most abundant genera representing each at least 1% of all assigned sequences (right panel). Data are means of replicate sequencing runs and cultures.

As shown in Fig 3, the composition of the microflora significantly shifted in the enrichment cultures, as compared to the initial compost sample. *Proteobacteria* were the predominant phylum (52%) in the compost which further contained 24% *Bacteroidetes*, 9% *Actinobacteria* with other phyla representing each less than 5%. Cultivation of the compost sample on the pretreated lignocellulosic substrates led to a significant reduction in the abundance of *Proteobacteria*. On the other hand, *Firmicutes* and *Chloroflexi* became more dominant after the enrichments. Interestingly, the abundance of other phyla was a function of the culture condition: *Bacteroidetes* were maintained at about the same level than in the initial compost sample in the solid culture, whereas sequences assigned to this phylum dropped down to 4% in the submerged cultures on LCB. This phylum regroups essentially anaerobic but relatively aerotolerant species, which may be able to survive in the static culture where anaerobic microenvironments could be maintained despite manual mixing and aeration. Also *Actinobacteria* were significantly enriched in the solid culture, but stayed at a low level in the submerged culture. The liquid LCB culture also led to an enrichment in the phyla *Deinococci* and *Gemmatimonadetes* which were almost undetectable in the initial compost sample and the enrichment under solid conditions.

At the genus level, *Thermomicrobium*, *Cohnella* and *Truepera* (phylum: *Deinococci*) were the most abundant genera in the LCB_l culture. They each make up about 5–7% of the total assigned bacterial sequences. In the LCB_s culture, *Cohnella* was also one of the dominant genera, together with *Conexibacter*, *Chitinophaga* and *Sphaerobacter*. However, the most frequently identified genus was *Rubrobacter*, with 9% of all bacterial sequences assigned to this genus in this culture (Fig 3).

### Analysis of biomass degradation potential

The biodegradation potential of each microcosm with respect to the original compost microflora was evaluated by the identification of CAZyme genes. For each metagenome, the analysis was performed on the reads on one hand, and on the assembled sequences, on the other, the latter reflecting CAZyme diversity. Sequences were screened for glycoside hydrolases, carbohydrate binding modules, polysaccharide lyases and auxiliary activities. Table 4 presents the averaged results, the distribution on replicate metagenomes is illustrated in S2–S5 Figs.

**Table 4:** Number of CAZyme hits on individual reads and assembled sequences.

	**reads**					
	GH	Cell	Hemicell	CBM	PL	AA
CI	2350	84	329	198	87	167
LCB_s	3357	179	570	295	112	219
LCB_l	1386	56	208	119	37	213
	**assembled**					
CI	389	13	69	20	14	25
LCB_s	351	29	36	34	9	26
LCB_l	127	4	12	10	3	14

Values are expressed as occurrence per 100 Mb for reads and per 10 Mb for assembled sequences. AA: auxiliary activities, CBM: carbohydrate binding module, GH: glycoside hydrolase, PL: polysaccharide lyase

A total of 954 CAZymes were detected in the initial compost sample, representing a frequency of 6.1 CAZymes per 100 kb. With a mean of 3.7 CAZymes per 100 kb, the frequency was lower in the LCB_l culture, but slightly higher (or about the same) in the LCB_s enrichment (6.6 / 100 kb). Over 600 sequences attributed to a glycoside hydrolase family were identified in the initial compost sample. When hit numbers were normalized by the total sequenced volume, the number of glycoside hydrolases (based on assembled sequences) did not change significantly in the LCB_s culture compared to the initial compost. There were about three times less glycoside hydrolases in the LCB_l enrichment, however, compared to the CI sample (Table 4 and S3 Fig). Upon closer inspection, 29 glycoside hydrolases assigned to families from which cellulase activity has been reported (families GH5, GH6, GH9, GH12, GH45, GH48 and GH74) were found in the LCB_s enrichment. This is twice as much as in the initial compost sample and more than seven times what found in the submerged LCB culture. 8% of all glycoside hydrolases were assigned to cellulases in LCB_s which is more than in the CI sample (3.5% and 2.6% in the CI and LCB_l sample, respectively) and also more than previously reported (5.6% [13]). The enrichment had different consequences on hemicellulase encoding genes: more representatives from families with hemicellulose activity (families GH10, GH11, GH26, GH30, GH43, GH51, GH53, GH54, GH67, GH113 and GH115) were identified in the LCB_s culture than in the initial compost metagenome, but they were less diverse. Hemicellulase enzymes tended to be less abundant in the LCB_l culture, but displayed clearly less diversity (S2 and S3 Figs). Four times less sequences of this category could be identified compared to the initial compost sample.

The total number of reads attributed to GH families with cellulase activities was increased after incubation in the LCB_s enrichment, especially due to a significant accumulation of genes encoding GH6, GH9 and GH48 enzymes (Table 4 and S4 Fig). Regarding diversity of potential cellulases in the LCB_s culture, an increase in members of families GH5, GH6 and GH48 was detected (S5 Fig). GH5 genes represented 5% of all GH assigned in the LCB_s culture, whereas it made up only 1% in the initial sample. Only two unique genes belonging to family GH12 could be detected from this enrichment, however. Representatives of families GH9 and GH74 were also present, but their number was not significantly different after enrichment. No hits were obtained for GH45 enzymes in any of the cultures analyzed. Interestingly, the liquid culture displayed significantly less genes encoding potential cellulases, and representatives from families GH6, GH48 and GH74 were almost completely absent, in contrast to the LCB_s culture.

The global diversity of potential hemicellulases did not increase in the enrichments. However, more unique representatives than in the original compost sample could be found in GH families with xylan and mannan-degrading activities (GH10, GH11 and GH26 in the both enrichment cultures and GH39 in the LCB_l culture). In contrast, less gene diversity was detected for families GH43, GH51, GH67 and GH115, with a large panel of hemicellulolytic activities, including β-xylosidases, xylanases, β-glucuronidases, α-L-arabinofuranosidases and arabinanases.

Concerning gene abundance, LCB_s displayed a higher number of hits in GH families with putative hemicellulose-degrading activities than the initial compost. In particular, enrichment could be observed for families GH30, GH51, where xylanase, β-xylosidase, endo-glucanase and arabinofuranosidase activities can be found, as well as families GH53 and GH115 (enzymes acting on galactane and glucuronic acid side chains). Family GH11 and GH26 enzymes (xylanases and mannanases) showed a slightly but significantly higher abundance, indicating a proliferation of hemicellulose-degraders during the enrichment. LCB_l had lost much of its genes assigned to families GH11, GH30, GH43 and GH67. However, the number of hits in GH10 families containing xylanase enzymes were not significantly altered in both types of enrichment. (see S4 Fig).

In parallel to the increase in putative cellulases in the LCB_s culture, cellulose binding domains belonging to families CBM2 also increased in this enrichment, but not in the liquid LCB culture. Interestingly, in the latter case, the number of genes assigned to CBM families binding to hemicellulose or with ubiquitous binding specificities, such as CBM22, CBM35 and CBM54 increased instead.

**Fig 4:**
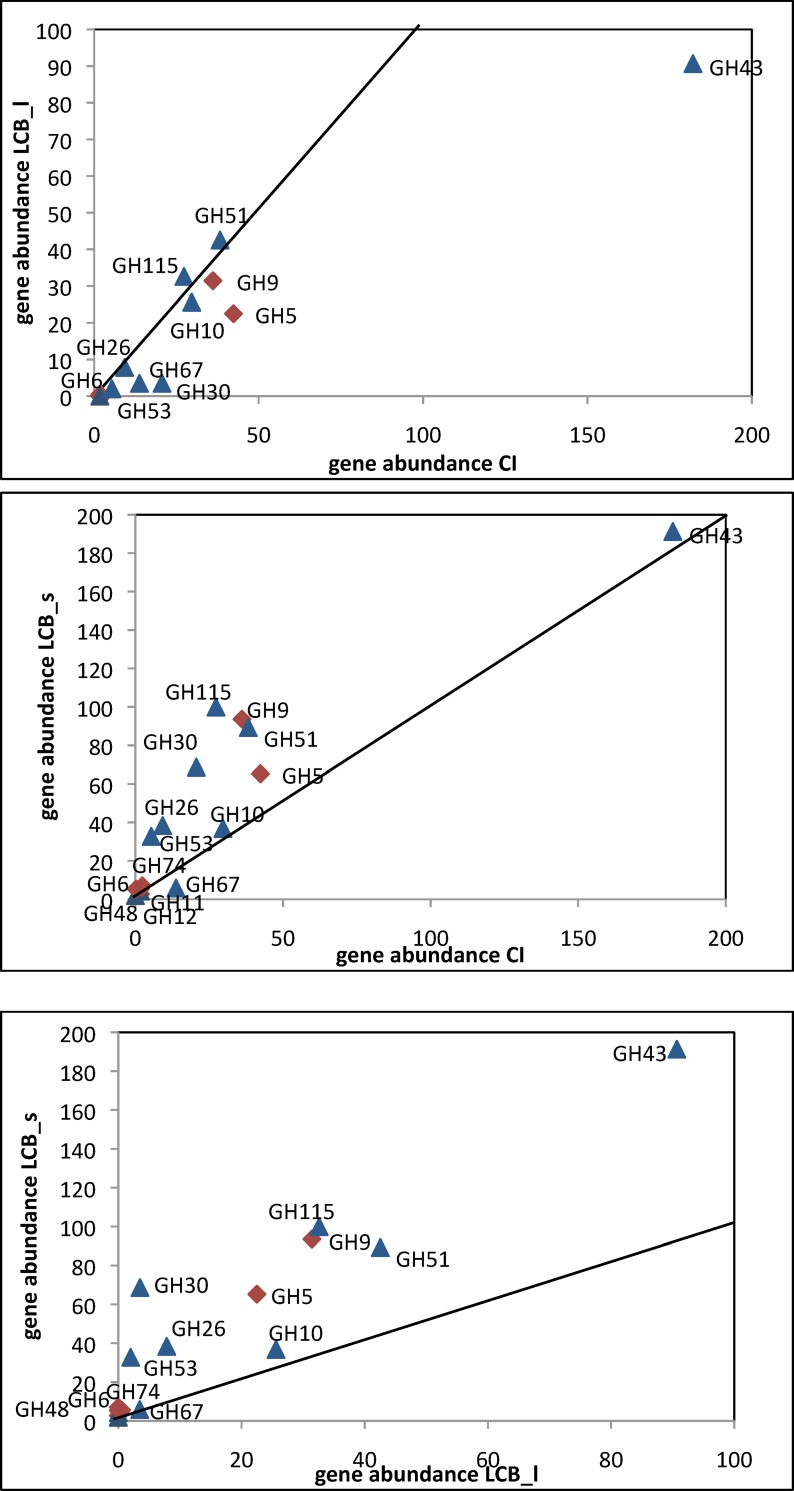
Abundance of cellulase and hemicellulase genes in CI, LCB_l and LCB_s metagenomes. The diagonals represent equal abundance. Red diamonds symbolyze cellulases and blue triangles hemicellulases. (a) CI versus LCB_l metagenome. GH11, GH12 and GH74 have low abundance in both metagenomes and are therefore not visible. (b) CI versus LCB_s (c) LCB_l versus LCB_s. GH11, GH12 and GH54 have too low abundance in both metagenomes to be visible. Data are normalized to 100 Mb.

A global picture of the cellulose and hemicellulose degradation potential of the three consortia is given in Fig 4. From pairwise comparisons, the very different properties of the LCB_s enrichment are clearly visible. Enrichment of putative cellulases from families GH5 and GH9 and hemicellulases from families GH26, GH30, GH51, GH53 and GH115 with respect to both the initial compost and the liquid culture are the most striking differences. On the contrary, none of the cellulose and hemicellulose degrading enzymes have been enriched in the LCB_l enrichment, the most dramatic difference to the initial compost being a loss in GH43 representatives.

Polysaccharide lyases which are involved in pectin degradation, i.e. from families PL4, have increased gene numbers in LCB_s culture. Both enrichments led to the same change in PL1 and PL9 diversity, the former family being decreased the latter increased.

More unique sequences were assigned to auxiliary activity families AA1 and AA7 (families of lignolytic enzymes) after enrichments, whereas less AA3 members, including cellobiose dehydrogenase and glucose oxidase activities, were detected in the solid culture compared to the initial sample. Two representatives of family AA10, regrouping bacterial lytic polysaccharide monooxygenases, could be detected in the assembled LCB_s metagenome, but none in the other samples. The general abundance of these genes was not significantly higher than in the initial compost metagenome, however. In general, AA family members were not enriched in the LCB_s culture. In the LCB_l culture, the global number of hits in the AA category was not significantly different from the CI sample, but this enzyme class was less diverse than the original sample.

### Phylogenetic distribution of cellulase and hemicellulase genes

**Fig 5:**
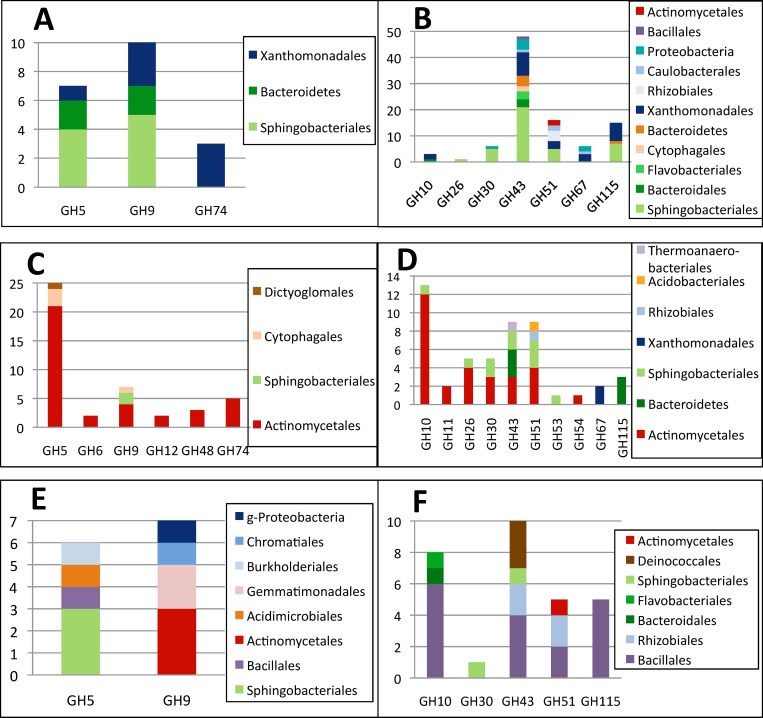
Assignment of glycoside hydrolase families with cellulase activities (panel A, C, E) and hemicellulase activities (panel B, D, F) to bacterial orders (or phylum level, if no order could be determined). Blastn analysis of contigs containing CAZymes of compost (A, B), LCB_s (C, D) and LCB_l (E, F) were realized against the non-redundant database and results visualized by MEGAN software. Y axis represents the total gene diversity (number of hits with assembled metagenomes).

Genes with probable cellulase and hemicellulase activities were analyzed for their phylogenetic origin. Concerning cellulases, the initial compost sample mainly contained genes assigned to families GH5 and GH9 that were most closely related to organisms belonging to the *Bacteroidetes* phylum (Fig 5A). The few GH74 genes detected in this sample could be attributed to the *Xanthomonadales*. Solid culture enrichment on LCB favored the accumulation of different cellulolytic organisms (Fig 5B), as most putative cellulases were most closely related to *Actinomycetales*, thus reflecting the observed change in total microflora composition. On the contrary, no enrichment of a specific bacterial order was found in the LCB_l culture. The only GH families with putative cellulase activities, GH5 and GH9, originated from several orders belonging to *Firmicutes*, *Actinobacteria*, *Proteobacteria*, *Bacteroidetes* and *Gemmatimonadetes* phyla.

GH families with hemicellulase activities from the compost sample were distributed among a more diverse spectrum of microorganisms, compared to cellulases. They were also most closely related to several orders from the *Bacteroidetes* phylum, but GH10, GH43, GH51 and GH115 were also attributed to *Xanthomonadales* and other *Proteobacteria*, as well as *Actinomycetales* and *Bacillales*. GH67 family members were exclusively related to *Xanthomonadales*. The LCB_s enrichment led to a higher diversity in GH families retrieved, but again mainly related to *Actinomycetales*. Interestingly, *Actinomycetales* from the studied microcosm did not seem to contain GH53, GH67 and GH115 hemicellulases. GH67 enzymes detected were stemming uniquely from *Xanthomonadales*, whereas GH115 enzymes were solely related to *Bacteroidetes*. In the LCB_l enrichment, the phylogenetic assignments of hemicellulase genes were very different than those of cellulase genes. Whereas the latter were attributed to distantly related orders, many of the former were similar to *Bacillales* and *Rhizobiales* genes. The observed accumulation of *Deinococci* in this enrichment was reflected by the detection of representatives of the GH43 family assignable to the *Deinococcales* order.

In summary, the results show a clear drift in microorganisms with putative (hemi)cellulose-degrading activities, from mostly *Bacteroidetes* in the original compost sample to *Actinomycetales* in the LCB_s culture. The non-accumulation of organisms with (hemi)cellulase activities in the LCB_l sample was demonstrated not only by the low number of hits in the corresponding GH families, but also by their rather low diversity and large distribution among different bacterial orders and phyla.

### General functional analysis

**Fig 6:**
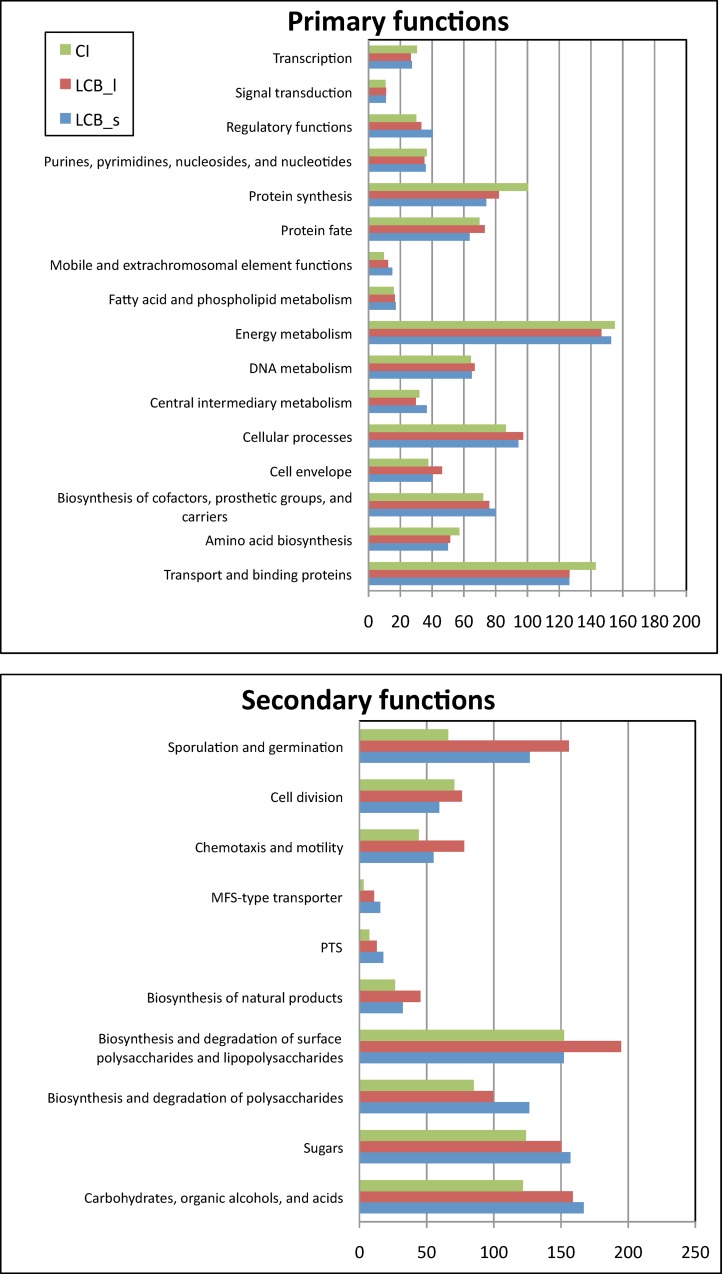
Functional analysis of metagenomes. Analysis was realized using reads and normalized to the total number of annotated genes in each meatgenome. In the upper panel representing primary functions, gene abundance is normalized to 10^3^ annotated genes. The lower panel presents secondary functions with gene numbers normalized to 10^4^ annotated genes.

In order to get a deeper insight into functional differences between the three samples, the metagenomes were screened for homologs and annotated using the Uniprot and TIGRFAM databases. Distribution of genes belonging to the different functional categories in the CI, LCB_l and LCB_s metagenomes are displayed in Fig 6. For some classes, remarkable changes in gene numbers occurred after enrichments: concerning the functional class of “transport and binding proteins”, the global number of genes was found to be decreased in LCB_l and LCB_s metagenomes. Interestingly, when looking into more detail, genes encoding transporters involved in sugar or oligosaccharide transport, such as MFS-type and PTS transporters showed the reverse tendency, i.e. their number was increased in enrichments, especially in the LCB_s metagenome (Fig 6, lower panel). In this sample, also more genes encoding ABC transporters were identified. The higher global number of genes in the CI sample in the “transport and binding proteins” functional class could be attributed to the increased abundance of genes involved in electron transport, porins and pathogenesis.

Noticeable differences in the abundance of annotated genes were in addition also visible in protein synthesis pathway. Transcription- and translation-related genes as well as genes involved in amino acid synthesis were reduced in the two enrichments. On the other hand, more genes involved in cellular processes were found in LCB_s and LCB_l enrichments, the secondary functions of this functional class with the most important gene abundance changes being sporulation and germination and chemotaxis and motility. Most of the genes found in the “sporulation and germination” class in the enriched metagenomes are annotated as being involved in cell wall modification, such as peptidoglycan synthetase or chitin deacetylase genes, as well as sporulation proteins and spore-cortex lytic enzyme-encoding genes. These changes suggest adaptation of the initial compost sample to the presence of new substrate and also better distribution of nutriments in the LCB_l culture. The change from a solid-state environment such as compost to a suspension culture is probably also at the origin of the higher abundance of genes found related to the cell envelope, and particularly to the “Biosynthesis and degradation of surface polysaccharides and lipopolysaccharides” which show higher gene numbers in the LCB_l enrichment compared to the two samples from the solid environment. Species with different cell wall composition might have proliferated in this culture, leading to an accumulation of glycosyltransferase, isoprenyl transferase, peptidoglycan-modifying protein or N-acetylglucosamine deacetylase genes, to give some examples. Genes encoding functions related to cell motility were also particularly increased in this culture, again indicating the proliferation of species adapted to the liquid environment.

A clear tendency could be observed concerning genes related to sugar, carbohydrate, organic acid and alcohol metabolism, as well as the metabolism of polysaccharides. After enrichment on lignocellulosic substrates, both in the liquid and the solid-state culture, increased gene numbers were detected as compared to the initial compost sample. Genes encoding permeases, sugar receptors and transporters were particularly enriched. The accumulation of polysaccharide modifying genes is particularly high in the LCB_s culture, being in good agreement with the observed accumulation of CAZyme-encoding genes.

## Discussion

Cost-competitive biofuel production still needs more efficient biomass degrading enzymes, even if important improvements have recently been achieved. Natural biodiversity is a large reservoir of lignocellulose-degrading enzymes and exposing microbial communities to industrially relevant substrates increases the probability of identifying efficient enzymes.

In this study, we compared two types of enrichment: a solid-state enrichment, imitating composting conditions, and an agitated liquid culture, such as it is often conducted in laboratories. In the first case, conditions were chosen to be close to compost facilities, by just increasing the C:N ratio and assuring optimal humidity. Cultures were static with only occasional manual mixing, mimicking the turning of a compost pile, which does not completely exclude the formation of anaerobic pockets in the culture. In the second case, a liquid suspension culture was set up, and incubated with agitation assuring good mixing of the inoculum with the substrate and minerals. Suspension and solid-state cultures were, however, both aerated when CO_2_ levels approached saturation, so that global oxygen availability was identical in the two cases.

Several previous studies conducted successful enrichments of CAZyme encoding genes on specific biomass such as wheat or rice straw, poplar or switchgrass, and some of them in liquid cultures [5,10,11,30]. In these studies, the incubation time was relatively short. In our study, changes in functional genes were not monitored during the incubation period and it is probable that changes began to occur already much earlier. However, to avoid analyzing a community hydrolyzing only “easily degradable” parts of the biomass, cultivation was prolonged to seven months and CO_2_ measurements indicated that the microbial community was still active. At that time point, a significantly different impact of the solid-state and the submerged culture set-ups on the microcosms was observed. But due to the single point analysis, the succession of microorganisms that had occurred during the enrichment period remains unknown. It is interesting to note, however, that in spite of the different incubation times in the present versus previous studies, some major features are similar: a high accumulation of *Actinobacteria* in compost-like enrichments or a higher proportion of *Firmicutes* and *Chloroflexi*, with a lower abundance of *Gemmatimodadetes* and *Trueperaceae* and nearly no *Actinobacteria* in a thermophilic liquid enrichment [9,10,14,30].

In contrast to previous studies, the present approach failed to enrich organisms with high biomass degradation potential in liquid cultures. It is possible that primary cellulose-degrading strains are efficiently enriched after short periods, whereas longer incubation eventually favors the growth of other strains able to feed on the easily digestible degradation products, that have been generated by truly cellulolytic strains. These organisms may have a faster growth rate, resist better to inhibitory molecules that accumulate in the medium over time or synthesize antimicrobial products, assuring them a dominant place in the consortium. On the contrary, incubation on the lignocellulosic substrate without a liquid phase does not allow dispersion of soluble degradation products, but probably directly benefits the organisms that are attached and liberate these products from the plant cell wall. In this case, cellulolytic organisms can be efficiently enriched. It would be interesting to confirm this hypothesis in future studies by analyzing several time points.

In nature, both bacteria and fungi play an important role in lignocellulose degradation, but bacteria generally dominate the thermophilic lignocellulose degrading consortia [31]. Although fungi are present throughout the composting process, recent studies suggest that thermophilic fungi are much less abundant than bacteria and that fungal biomass degradation activity is limited during the thermophilic phase [32,33]. The present study therefore focused on the prokaryotic population and the DNA extraction protocol used aimed at isolating prokaryotic DNA. Metagenome analysis confirmed that only a low percentage of eukaryotic sequences were obtained (about 2%). In addition, CAZymes of fungal origin in the initial compost and LCB_s metagenomes were scarce (between 2 and 6%) and in the LCB_l metagenomes, no fungal CAZymes were detected at all. Furthermore, no genes encoding CAZymes with only eukaryotic representatives, such as those from families CBM1, GH7 or AA9 were detected in any of the metagenomes. In the past, the contribution of thermophilic or thermotolerant fungi to biomass degradation in compost has rarely been addressed and would constitute an interesting subject of future studies.

Concerning the bacterial population, the initial compost sample contained a majority of *Proteobacteria* and nearly 25% *Bacteroidetes*. A high proportion of *Proteobacteria* is frequently found in mesophilic lignocellulose-degrading microcosms, such as mangrove [34], soil [16] or sugarcane bagasse [15], but also in the initial phases of composting [35]. In our case, the compost was taken at the thermophilic stage, indicating that a high number of *Proteobacteria* were able to survive under these conditions. Also *Bacteroidetes* were abundant in this sample, and members of this phylum are known plant cell wall polysaccharide degraders, especially in termite, herbivore and human gut microbiota [4,36]. Analysis of the phylogenetic origin of glycoside hydrolases in the CI sample confirmed that a majority of cellulases and hemicellulases were most similar to genes from this phylum. Interestingly, nearly no *Firmicutes* were present in the initial compost, whereas about 10 to 15% were found in the initial phase of a zoo compost [35]. *Actinobacteria* generally make up about 7–8% in such consortia, and this was also the case in the compost used here.

Enrichment in liquid culture led to accumulation of different phyla which were less often associated with plant cell wall degradation in the past. In particular, the accumulation of the genus *Truepera* belonging to the *Deinococcus* phylum or *Gemmatimonadetes* could be detected. [10]Sequenced species of these families also contain some CAZyme-encoding genes in their genome, and in the present case, a gene encoding a family GH9 enzyme most closely related to a gene product from *Gemmatimonadales* and a family GH43 encoding gene related to genes from the *Trueperaceae* family were retrieved from the translated metagenome. The high proportion of (hemi)cellulases related to *Bacillales* could be a consequence of the partly anaerobic conditions in the suspension culture, in spite of continuous shaking. An important accumulation of *Bacillales* (in particular *Paenibacilli*) was also observed on switchgrass-adapted cultures [10].

The thermophilic enrichment on solid LCB led to a large increase in *Actinobacteria*, a phenomenon that was also observed in a similar experiment using switchgrass or rice straw as a substrate [9,14,30]. The importance of this phylum is confirmed by the fact that nearly all cellulases and hemicellulases were assigned to this phylum in the LCB_s enrichment. After enrichment on rice straw, Wang et al. [14] found that, in particular, cellobiohydrolases of family GH48 originated from *Actinobacteria*. In our LCB_s enrichment, the three putative LPMOs (family AA10) were also assigned to organisms from this phylum (*Thermobispora* and *Streptomyces*). As shown in the sequence section of CAZy, sequenced genomes of *Thermobispora bispora* and *Microbacterium testaceum* reveal a large panel of glycoside hydrolases, including putative cellulases from families GH5, GH6, GH9, GH12, GH48 and GH74 as well as hemicellulases GH10, GH11, GH42, GH51, GH53 and GH115. It is thus possible that the glycoside hydrolases found in the LCB_s enrichment originate from one or just a few dominant strains.

Only two main GH families with putative cellulase activities, namely GH5 and GH9, were detected in CI and LCB_l. These two families are frequently detected in metagenomes from lignocellulose-degrading consortia [13,15]. On the contrary, GH6, GH12, GH45 and GH48 are much less often retrieved in such analyses [34]. In our case, GH6, GH12 and GH48 were only detected in the LCB_s enrichment. The higher diversity of putative cellulases in this microcosm might be one of the reasons for the high CMCase activity detected in this case.

Concerning xylanase activity, the highest value was obtained with the CI microcosm. Surprisingly, in this metagenome, the number of putative xylanase genes from families GH10 and GH11 was not significantly different from those retrieved in the enrichments. However, this activity can also be due to enzymes from other GH families, such as GH30, GH43 and GH51. The CI metagenome indeed contained a high number of genes encoding GH43 enzymes. Also in this case, the significantly higher diversity of hemicellulases (number of hits in the assembled metagenome) compared to the enrichments could be at the origin of the high xylanase activity detected.

## Conclusions

Adaptation of a compost sample to pre-treated, industrially relevant lignocellulosic substrates led to significant changes in the metaCAZome. The study revealed that the set-up of such adaptation experiments has a major impact on the resulting consortia and their functional capacities. In addition, analysis of the cellulolytic potential of this metagenome highlighted the importance of *Actinobacteria* in the thermophile solid enrichment. A high diversity in putative cellulases, probably encoded by only a few species of this phylum, seems to be an important factor for efficient cellulose degradation. Pure strains from the two microcosms are currently being isolated and their characterization and genome sequencing should further confirm the role of these organisms in lignocellulose degradation.

## Supporting Information

S1 Fig Rarefaction curve of the original compost metagenome CI, and LCB_S1, LCB_S2, LCB_L1, LCB_L2 and LCB_Lr metagenomes realized with MG-RAST. (TIF)

S2 Fig Bootstrap analysis of read numbers with hits in the respective CAZy families and cellulase end hemicellulase subsets (for further detail see text) for each 454 semi-run. CI = initial compost; L1, L2 = liquid enrichment metagenomes; Lb = replicate liquid enrichment metagenome; S1, S2 = solid enrichment metagenomes. A: glycoside hydrolases, B: carbohydrate binding modules, C: polysacchraide lyases, D: auxiliary activities, E: cellulases; F: hemicellulases
(TIF)

S3 Fig Bootstrap analysis of contig numbers with hits in the respective CAZy families and cellulase end hemicellulase subsets (for further detail see text) for each 454 semi-run. CI = initial compost; L1, L2 = liquid enrichment metagenomes; Lb = replicate liquid enrichment metagenome; S1, S2 = solid enrichment metagenomes. A: glycoside hydrolases, B: carbohydrate binding modules, C: polysacchraide lyases, D: auxiliary activities, E: cellulases; F: hemicellulases
(TIF)

S4 Fig Boxplots of Ф^2^ contributions of CAZymes to the total number of reads containing a selection of 32 CAZy families involved in lignocellulose degradation, ordered by decreasing importance of contribution. (PPTX)

S5 Fig Boxplots of Ф^2^ contributions of CAZymes to the total number of contigs containing a selection of 32 CAZy families involved in lignocellulose degradation, ordered by decreasing importance of contribution. (PPTX)

S1 Table Statistics of assembly using Abyss, CLC or Metavelvet algorithms. (DOCX)
