# Supplementary figures and images for: Composting-Like Conditions Are More Efficient for Enrichment and Diversity of Organisms Containing Cellulase-Encoding Genes than Submerged Cultures
Source: PLoS One. 2016 Dec 9;11(12):e0167216. doi: 10.1371/journal.pone.0167216 (PMC5147896; doi:10.1371/journal.pone.0167216)

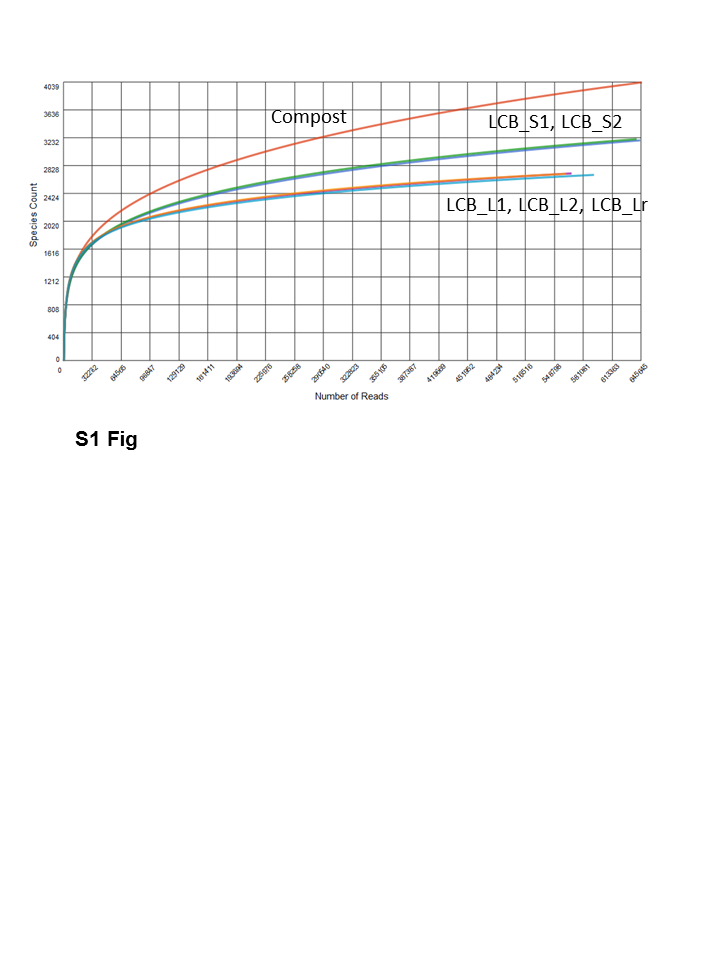

Supplement: S1 Fig — (TIF) [file pone.0167216.s001.tif]

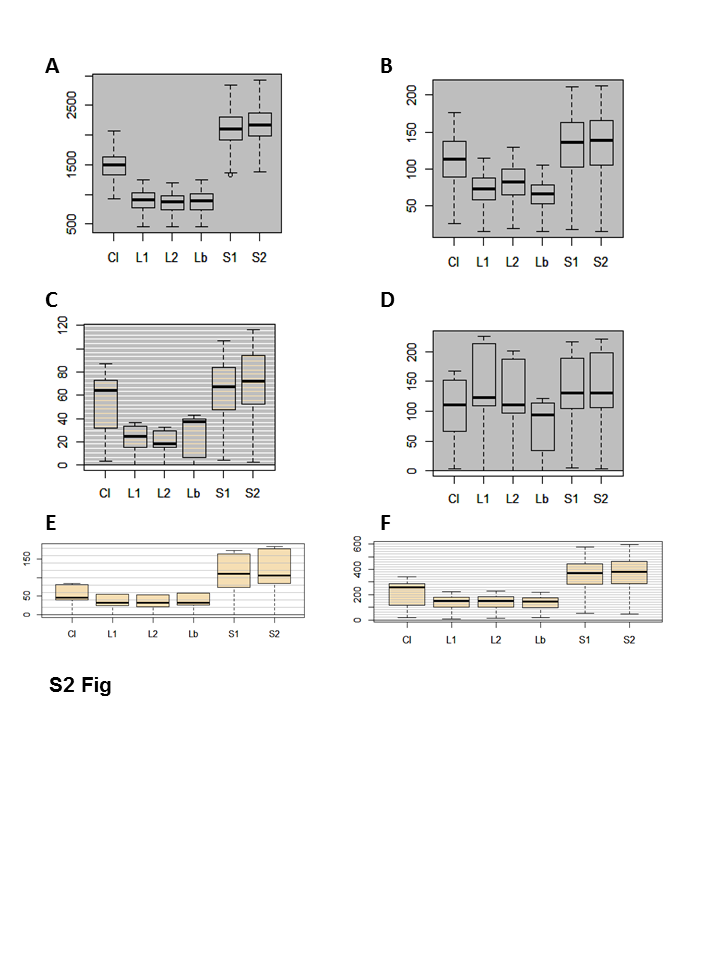

Supplement: S2 Fig — CI = initial compost; L1, L2 = liquid enrichment metagenomes; Lb = replicate liquid enrichment metagenome; S1, S2 = solid enrichment metagenomes. A: glycoside hydrolases, B: carbohydrate binding modules, C: polysacchraide lyases, D: auxiliary activities, E: cellulases; F: hemicellulases (TIF) [file pone.0167216.s002.tif]

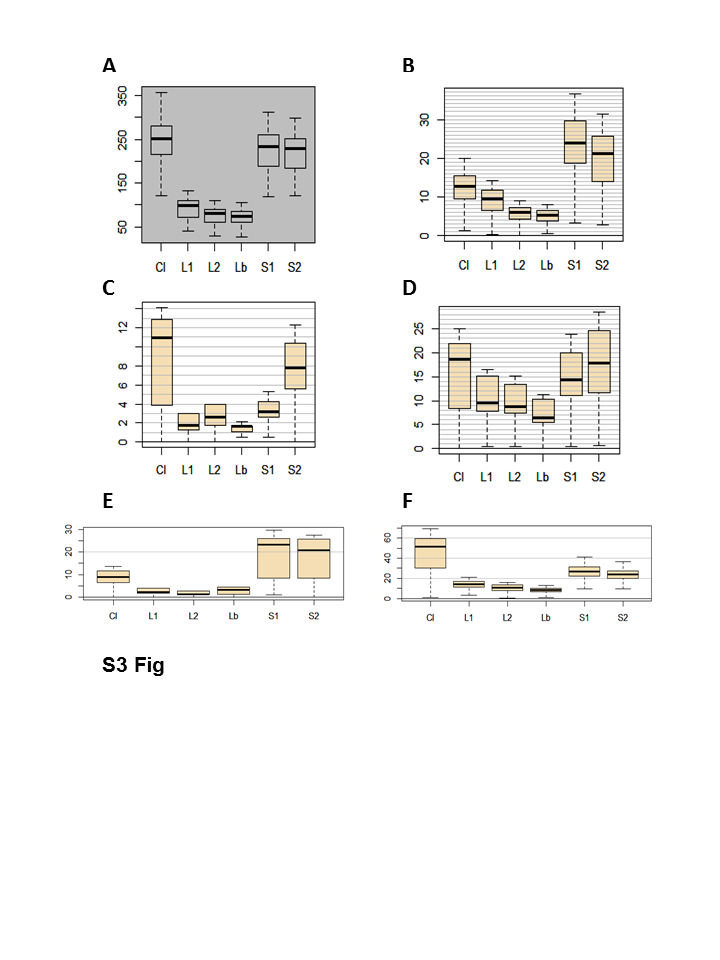

Supplement: S3 Fig — CI = initial compost; L1, L2 = liquid enrichment metagenomes; Lb = replicate liquid enrichment metagenome; S1, S2 = solid enrichment metagenomes. A: glycoside hydrolases, B: carbohydrate binding modules, C: polysacchraide lyases, D: auxiliary activities, E: cellulases; F: hemicellulases (TIF) [file pone.0167216.s003.tif]

## Slide 1
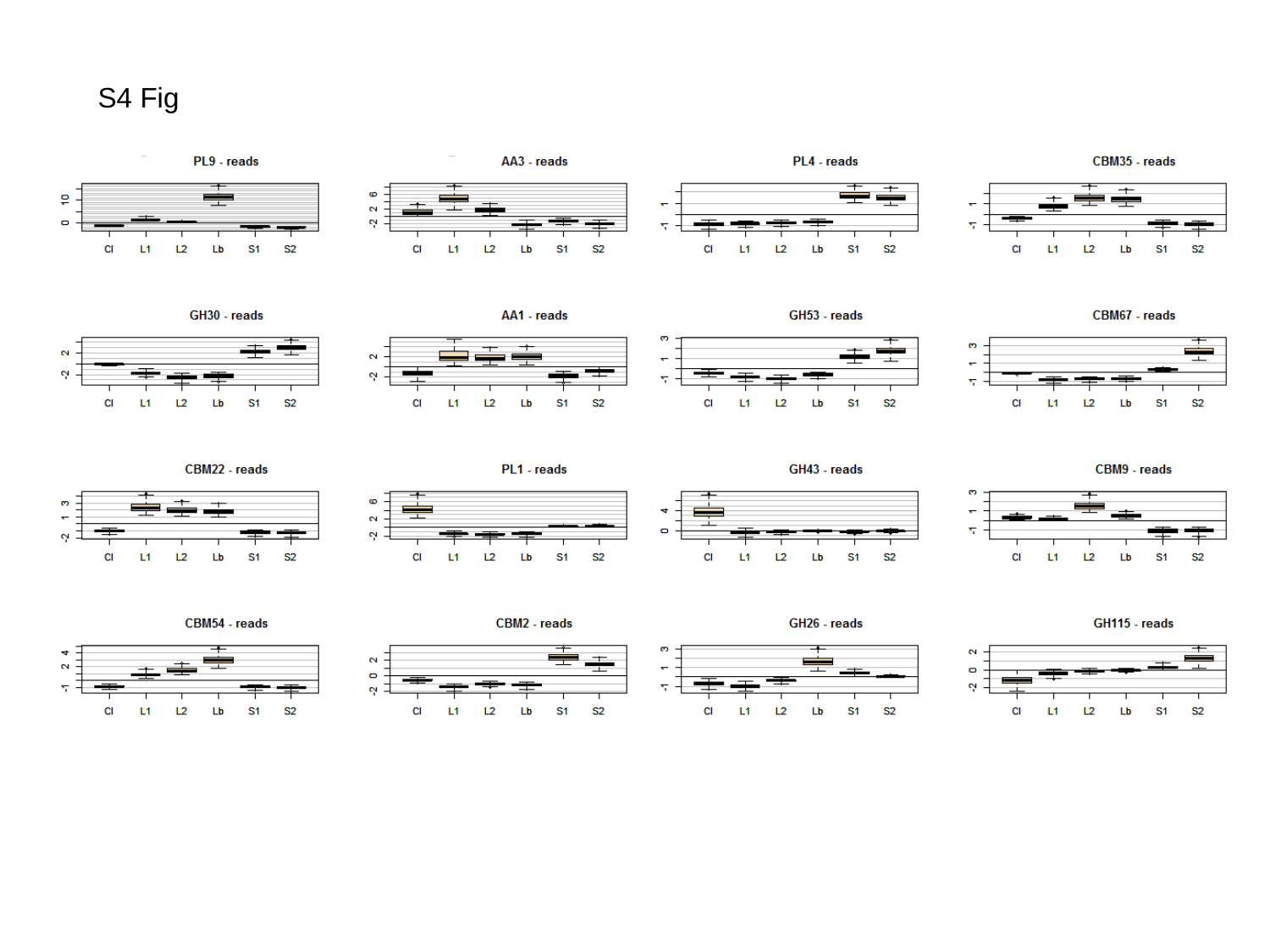

S4 Fig

## Slide 2
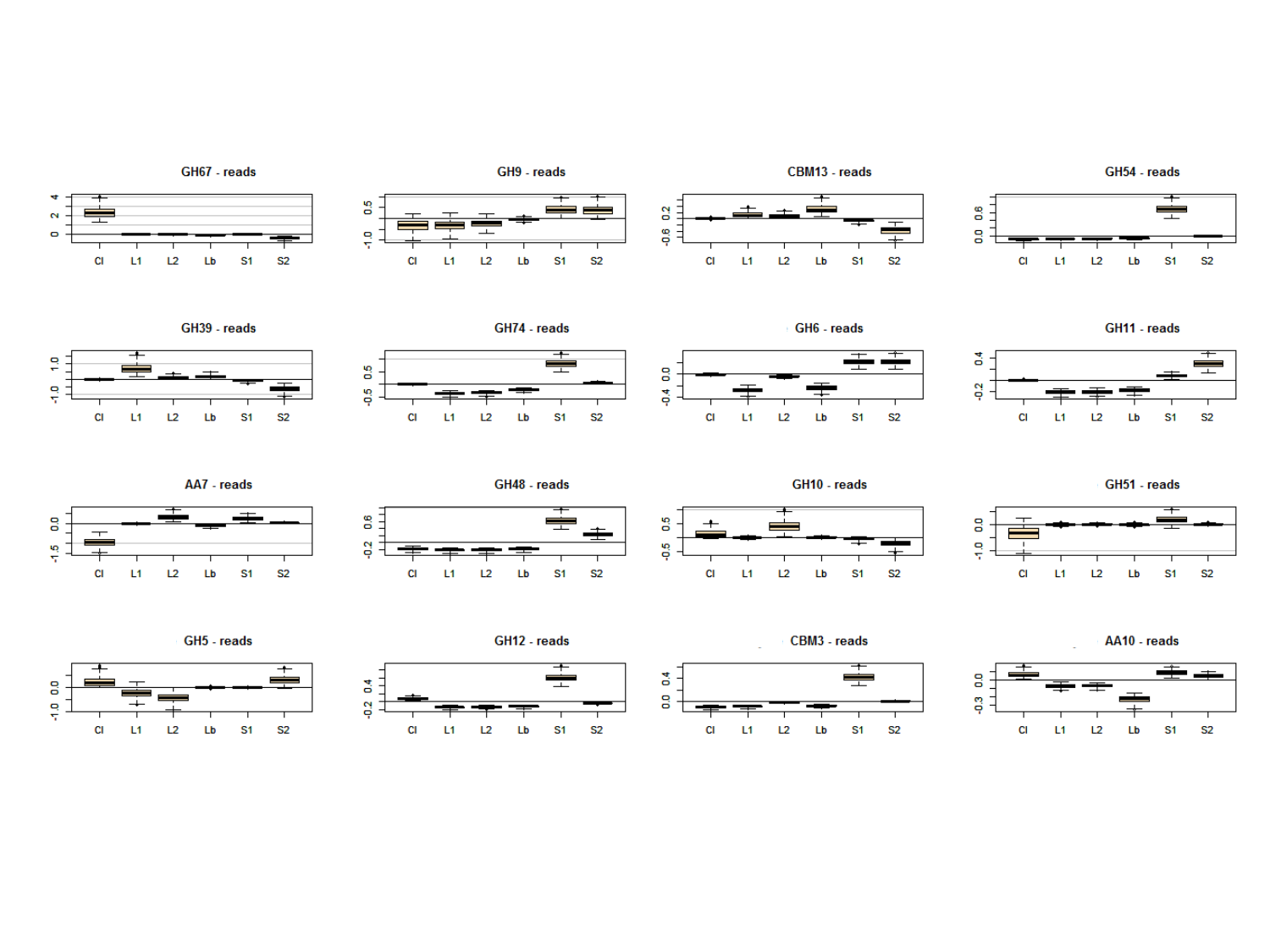

Supplement: S4 Fig — (PPTX) [file pone.0167216.s004.pptx]

## Slide 1
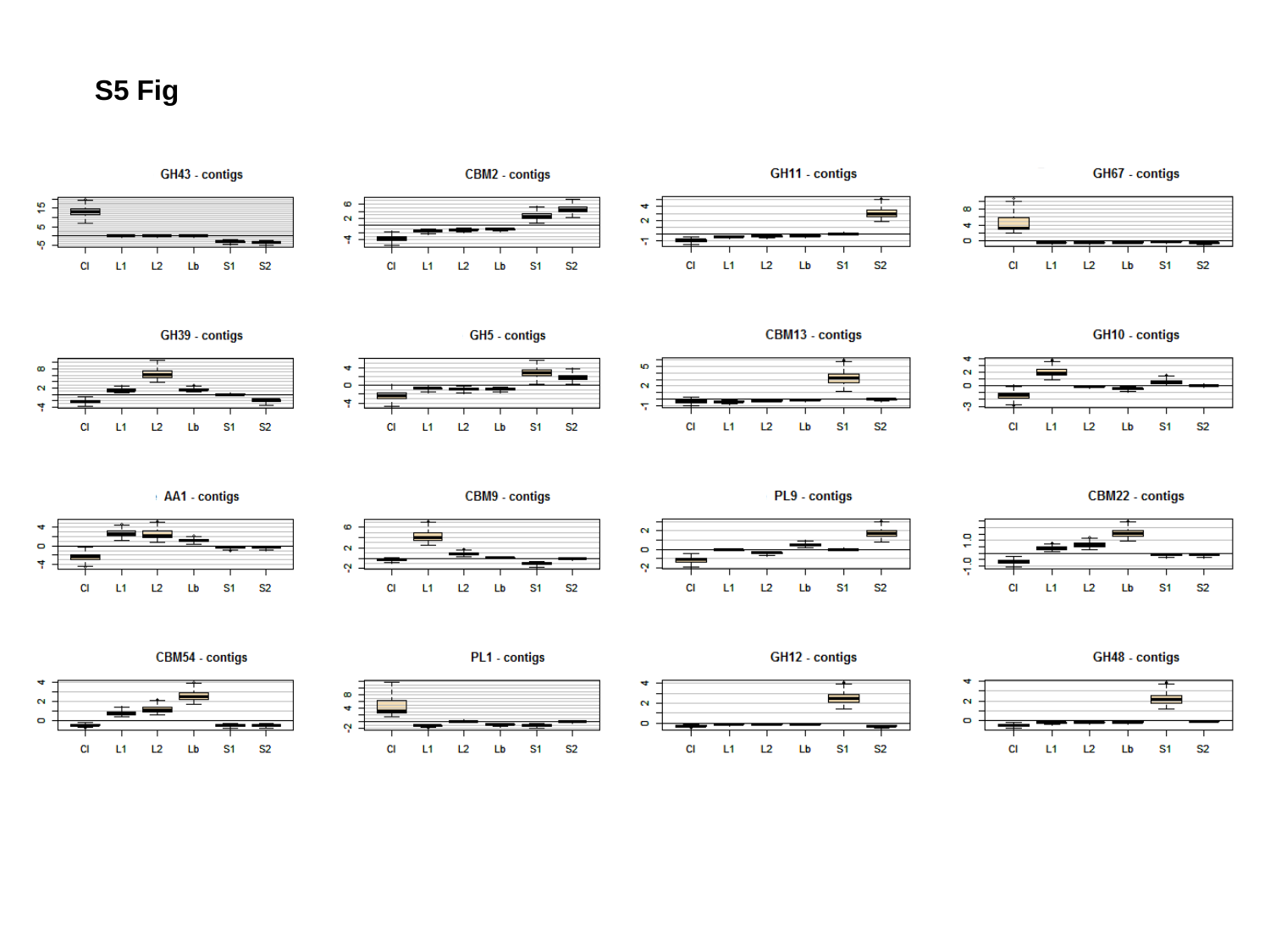

S5 Fig

## Slide 2
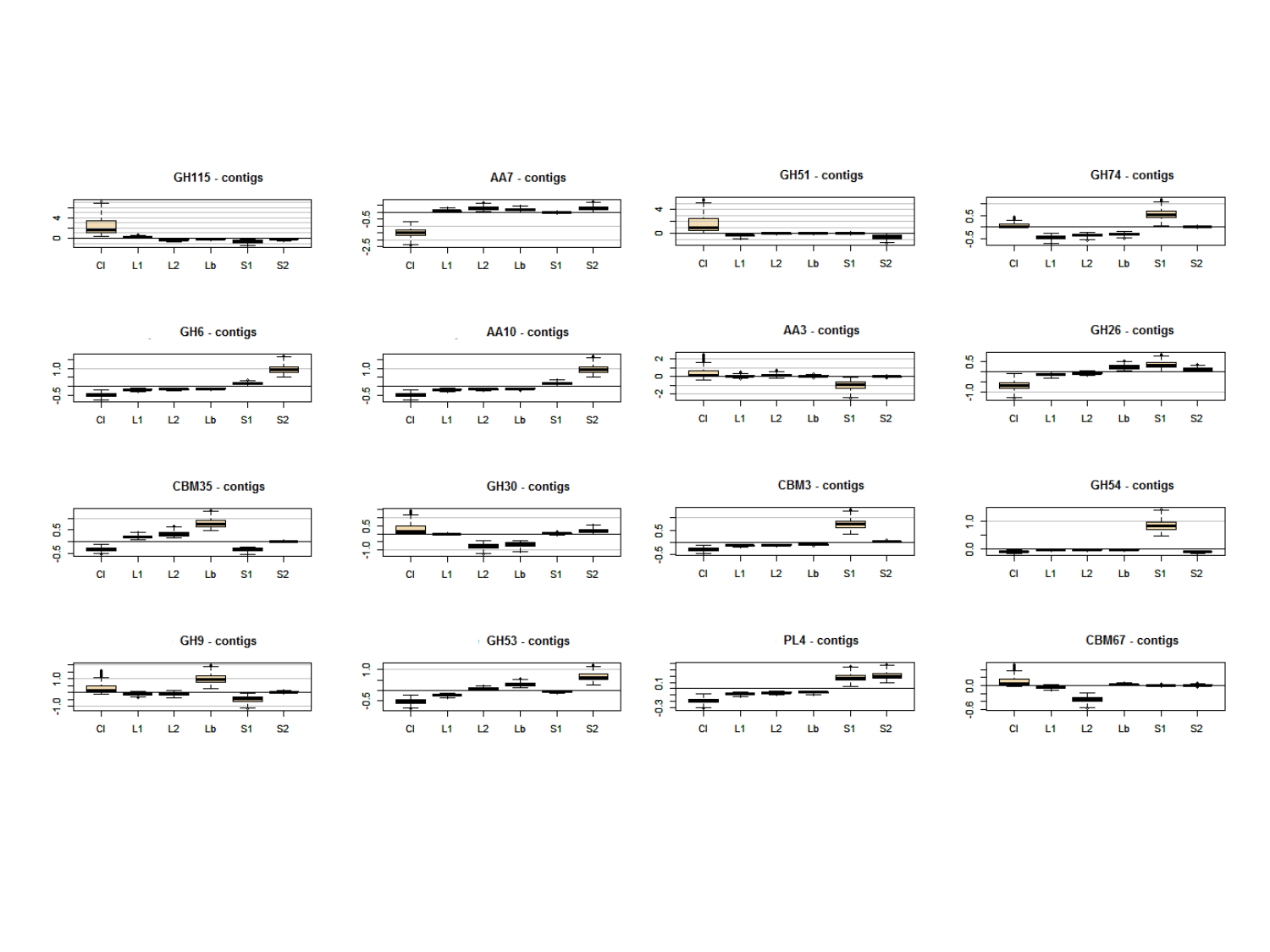

Supplement: S5 Fig — (PPTX) [file pone.0167216.s005.pptx]
