# Supplementary material for: Composting-Like Conditions Are More Efficient for Enrichment and Diversity of Organisms Containing Cellulase-Encoding Genes than Submerged Cultures
Source: PLoS One. 2016 Dec 9;11(12):e0167216. doi: 10.1371/journal.pone.0167216 (PMC5147896; doi:10.1371/journal.pone.0167216)
Supplement: S1 Table — (DOCX) [file pone.0167216.s006.docx]

**S1 Table :** Statistics of assembly using Abyss, CLC or Metavelvet algorithms.

|  | N contigs | Max contig length | Total length | N50 | Overassembly |
| --- | --- | --- | --- | --- | --- |
| CI | 12159 / 20617 / 16617 | 5123 / 8533 /3890 | 9695523 / 24116528 / 13004700 | 802 / 1235 / 777 | 8,33 / 33,33 / 3,03 |
| LCB_L1 | 12978 / 13192 / 18202 | 18184 / 50546 / 4762 | 19029378 / 28714832 / 16301594 | 1849 / 2787 / 918 |  |
| LCB_L2 | 12209 / 12829 / 18720 | 20684 / 46104 / 5631 | 19210184 / 29868261 / 17342919 | 2025 / 2994 / 961 |  |
| LCB_Lr | 11072 / 10111 / 14327 | 12682 / 30160 / 5199 | 15308629 / 22117939 / 13000556 | 1623 / 2708 / 936 |  |
| LCB_S1 | 12916 / 19917 /16701 | 8937 / 16159 / 3880 | 12331948 / 28463476 /14192262 | 992 / 1630 / 852 |  |
| LCB_S2 | 12114 / 18646 / 15547 | 13508 / 18297 / 4231 | 11470086 / 27008577 / 13279874 | 975 / 1645 / 854 |  |
